# Local staging with multiparametric MRI in daily clinical practice: diagnostic accuracy and evaluation of a radiologic learning curve

**DOI:** 10.1007/s00345-018-2295-6

**Published:** 2018-04-21

**Authors:** B. H. E. Jansen, F. H. K. Oudshoorn, A. M. Tijans, M. J. Yska, A. P. Lont, E. R. P. Collette, J. A. Nieuwenhuijzen, A. N. Vis

**Affiliations:** 1 VU University Medical Center, Amsterdam, The Netherlands; 2 Maasstad Ziekenhuis, Rotterdam, The Netherlands; 3 Meander Medisch Centrum, Amersfoort, The Netherlands

**Keywords:** Prostatic neoplasms, Magnetic resonance imaging, Neoplasm staging, Learning curve

## Abstract

**Purpose:**

To estimate the diagnostic accuracy of multiparametric MRI (mpMRI) for the detection of locally advanced prostate cancer (T-stage 3–4) prior to radical prostatectomy, in a multicenter cohort representing daily clinical practice. In addition, the radiologic learning curve for the detection of locally advanced disease is evaluated.

**Methods:**

Preoperative mpMRI findings of 430 patients (2012–2016) were compared to pathology results following radical prostatectomy. The diagnostic accuracy (sensitivity, specificity, PPV, and NPV) for the detection of locally advanced disease was calculated and compared for all years separately, to evaluate the presence of a radiological learning curve.

**Results:**

Of all 137 patients with locally advanced disease, 62 patients were preoperatively detected with mpMRI [sensitivity 45.3% (95% CI 36.9–53.6%), specificity 75.8% (CI 70.9–80.7%), PPV 46.6% (CI 38.1–55.1%), and NPV 74.7% (CI 69.8–79.7%)]. The diagnostic accuracy did not improve significantly over time (sensitivity *p *= 0.12; specificity *p *= 0.57).

**Conclusions:**

In daily clinical practice, the diagnostic accuracy of mpMRI for the detection of locally advanced prostate cancer remains limited. It, therefore, seems questionable whether mpMRI is adequate to guide preoperative decision-making. No significant radiologic learning curve for the detection of locally advance disease was observed.

## Introduction

Prostate cancer (PCa) is the most common cancer in men of older age in Western countries [1]. Accurate staging of the primary tumor is of vital importance, as the distinction between organ-confined disease (T-stage 1–2) versus locally advanced tumors (T3–4) influences both prognosis [2] and treatment planning [3].

The main therapeutic approaches for PCa include radical prostatectomy and radiotherapy [3]. When considering a radical prostatectomy, the presence of locally advanced disease warrants a concomitant extended pelvic lymph node dissection (ePLND), as there is an increased risk of lymph node metastasis [3–5]. In addition, local tumor stage guides surgical planning regarding the preservation of the neurovascular bundle. Nerve-sparing surgery is generally restricted to patients with organ-confined disease. Extension of PCa outside the prostatic capsule requires dissection of the neurovascular bundle, for nerve-sparing surgery would increase the risk of positive surgical margins [3, 4, 6]. The assessment of the local tumor stage is similarly important when radiotherapy is chosen as treatment and guides decisions on radiation dose, radiation template, and adjuvant therapies [3, 7].

For assessment of the local tumor stage, routine diagnostics (i.e., digital rectal examination, serum prostate-specific antigen (PSA) level, transrectal ultrasound, and biopsy Gleason score [3, 8]) are insufficient [9]. When combining these clinical parameters into predictive nomograms (e.g., the Partin Tables), staging accuracy increases, but remains imperfect [10, 11].

To overcome this diagnostic shortcoming, multiparametric magnetic resonance imaging (mpMRI) is increasingly deployed. mpMRI is an imaging technique that combines different (functional) imaging sequences, generating improved detection, and localization of malignant lesions. Although mpMRI presents promising detection of PCa [12], accurate assessment of the tumor stage is still imperfect. In a recent meta-analysis, the sensitivity of mpMRI for overall T3 detection reached 61% only (95% CI 54–67%) [13].

A concern regarding mpMRI is the considerable inter-observer variability [14, 15]. This problem might be due to different experience of radiologists with mpMRI, as a marked radiologic learning curve was demonstrated [16–19]. The presence of such learning curve, however, is studied mainly for primary detection of PCa. Research specifically evaluating the existence of a radiological learning curve for correct staging of PCa is scare, focusing mainly on endorectal MRI [18]. In the cited meta-analysis, the effect of radiologists’ experience on diagnostic accuracy was evaluated, but the results were inconclusive [13].

In this study, we aimed to estimate the diagnostic accuracy of mpMRI for the detection of locally advanced PCa stages (pT3–4) prior to radical prostatectomy, in a multicenter, real-life clinical cohort of patients. We additionally assessed the diagnostic accuracy over time, evaluating the existence of a radiologic learning curve.

## Materials and methods

### Subjects

For this study, 430 concurrent patients were retrospectively analyzed. Inclusion criteria were histologically confirmed prostate adenocarcinoma, for which a robot-assisted laparoscopic radical prostatectomy (RARP) and preoperative mpMRI were performed. Both MRI acquisitions made before or after prostate biopsy were considered eligible for inclusion, as in both scenarios, staging information is provided. The indication to perform an RARP as well as an mpMRI was made according to the locally valid clinical guidelines [3, 20]. These guidelines recommend mpMRI for intermediate- and high-risk patients [3] and when ‘clinically relevant for therapy planning’—explicitly mentioning decisions regarding nerve-sparing surgery [20]. In what exact scenarios an mpMRI is clinically relevant is left to the urologists’ discretion.

Patients were included from 2012 until 2016, in three hospitals in The Netherlands (VU University Medical Center, Amsterdam; Maasstad Ziekenhuis, Rotterdam; Meander Medisch Centrum, Amersfoort). For all participants, demographic and clinical data were retrieved (e.g., age, clinical stage, prostate biopsy results, and recent PSA).

### Imaging protocol and analysis

All institutions used three Tesla MRI scanners (GE^®^, Siemens^®^). The imaging protocol included T1-weighted, T2-weighted, diffusion weighed, and dynamic contrast-enhanced imaging. No endorectal coils were used. Per hospital, mpMRI interpretation was done by two to three radiologists dedicated to prostate mpMRI reading. As our series report on staging in daily clinical practice, the radiologists were not blinded to available clinical information and revisions of mpMRI acquisitions from referred patients were not standardly performed. During the course of this study, the use of standardized reporting for mpMRI became in use (PI-RADS v1 [21]; PI-RADS v2 [22]), providing guidelines for assigning rT3–4 stages on mpMRI.

### Pathologic analysis

RARP specimens were processed according to clinical routine [3] in the participating hospitals by dedicated uro-pathologists. No centralized review of the analyses was performed. Specimens were fixated with formaldehyde (10%) and the apex and base removed. The mid part of the specimen was cut perpendicular to the urethra in 4 mm slices; the apex and base were cut in sagittal fashion. The resulting slices were processed after sectioning in quadrants. Pathology reporting included histopathologic cancer type, Gleason score, and explicit notation of the presence or absence of any form of local tumor advancement (pT3a, pT3b, and pT4).

### Statistics

Overall detection of malignancy was calculated (sensitivity). When a PI-RADS classification was given, scores 4 and 5 were considered a positive test result. Radiological T-stage (rT) based on mpMRI was compared to the pathological T-stage (pT). Sensitivity, specificity, positive predicting value (PPV), and negative predicting value (NPV) of mpMRI were calculated for locally advanced disease (pT3–4). To examine if the accuracy of mpMRI was different in patients with a high risk of locally advanced disease, all patients with a Gleason score ≥ 8 and/or a PSA of ≥ 20 ng/ml were identified. The diagnostic accuracy in this high-risk group was compared to the accuracy in the lower risk group (Gleason score 6–7; PSA less than 20 ng/ml).

Finally, the diagnostic accuracy was analyzed for all years (2012–2016) separately, to evaluate the presence of a radiological learning curve. To overcome small sample sizes per year, an extra analysis of diagnostic accuracy based on the first and second half of the inclusions per hospital was performed. Differences in diagnostic performance were checked for statistical significance (*p *< 0.05) using the *χ*^2^ test.

## Results

An overview of the patients’ characteristics is presented in Table 1. A PI-RADS classification was given in 60.0% of all cases (rising from 0% in 2012 to 65.1% in 2016). Pathology analysis following radical prostatectomy revealed extra-prostatic extension (pT3a) in 76 (18%) patients, seminal vesical invasion in 57 (13%) patients, and advancement of the tumor into adjacent structures (pT4) in 4 (1%) patients.

**Table 1:** Characteristics of included patients

Patient characteristics and pathology results	
Age (years)	66 (61–69)
PSA (ng/ml)	9.2 (6.2–14.9)
Prostate volume (ml)	48 (37–65)
Number of biopsy cores	8 (8–10)
% of positive biopsy cores	49.1
Gleason score (biopsy)	
6	156 (36%)
7	182 (42%)
8	59 (14%)
9	27 (6%)
10	5 (1%)
Pathology (prostatectomy specimens)	
pT0	4 (1%)
pT2a	40 (9%)
pT2b	9 (2%)
pT2c	240 (56%)
pT3a	76 (18%)
pT3b	57 (13%)
pT4	4 (1%)

Median and interquartile ranges; number and percentages of total

The presence of malignancy was correctly detected by mpMRI in *n *= 358 patients (sensitivity 84.0%, CI 80.6–87.5%). In Table 2, the findings on preoperative mpMRI (rT) and concurrent RARP pathology results (pT) are depicted.

**Table 2:** Cross-tabulation of pathological tumor stage (pT) and radiological tumor stage (rT) for 430 patients undergoing mpMRI and robot-assisted radical prostatectomy (RARP)

	Pathological tumor stage						
	pT0	pT2	pT3 total	pT3a	pT3b	pT4	Total
Radiological tumor stage							
rT0/rTx	2	56	**12**	**9**	**3**	**0**	70
	0%	13%	**3%**	**2%**	**1%**	**0%**	16%
rT2	2	162	**59**	**32**	**27**	**4**	227
	0%	38%	**14%**	**7%**	**6%**	**1%**	53%
rT3 total	*0*	*70*	62	x	x	0	132
	*0%*	*16%*	14%			0%	31%
rT3a	*0*	*68*	x	33	16	0	117
	*0%*	*16%*		8%	4%	0%	27%
rT3b	*0*	*2*	x	2	11	0	15
	*0%*	*0%*		0%	3%	0%	3%
rT4	*0*	*1*	0	0	0	0	1
	*0%*	*0%*	0%	0%	0%	0%	0%
Total	4	289	133	76	57	4	430
	1%	67%	31%	18%	13%	1%	100%

Indicated in bold are cases of radiologic understaging, with potential oncologic hazard. Indicated in italic are cases with radiologic overstaging, influencing the decision to perform nerve-sparing surgery

mpMRI detected 62 out of 137 patients with locally advanced disease (pT3–4), resulting in a sensitivity of 45.3% (CI 36.9–53.6%), specificity 75.8% (CI 70.9–80.7%), PPV 46.6% (CI 38.1–55.1%), and NPV of 74.7% (CI 69.8–79.7%). Sensitivity in the group with high risk of locally advanced disease (*n *= 133) was 49.2% (CI 36.4–61.9%) versus 42.3% (CI 31.4–53.3%) in the lower risk group (*n *= 297) (*p *= 0.49). Specificity was 73.0% (CI 62.9–83.1%) and 76.3% (CI 70.6–82.0), respectively.

Radiologic understaging (i.e., the failure to detect locally advanced disease) occurred in *n *= 75 cases (54.7% of all patients with pT3–4). In *n *= 30 of these patients, no ePLKD was performed (21.9% all patients with pT3–4), and in *n *= 41 patients, complete nerve preservation was performed (29.9% of all pT3–4). This reveals the potential undertreatment associated with incorrect radiologic staging. Radiologic overstaging (incorrect detection of locally advanced disease) was present in *n *= 70 cases (23.9% of patients with pT0–2). In *n *= 16 of these cases, no form of nerve-sparing was performed (5.5% of all patients with organ-confined disease), revealing potential overtreatment.

**Fig. 1:**
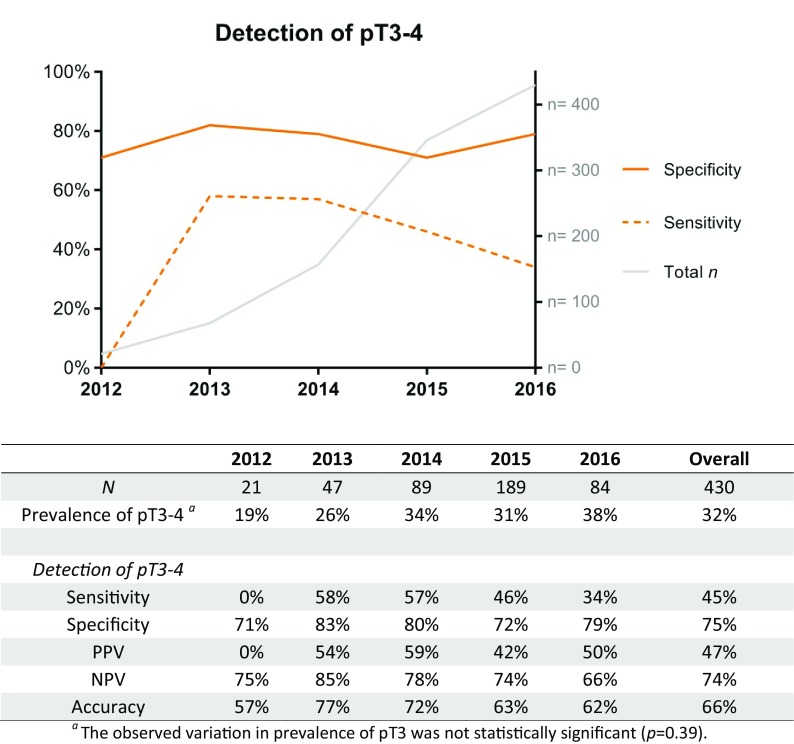
Number of included mpMRI procedures between 2012 and 2016 and the radiologic detection of locally advanced disease (pT3–4)

In Fig. 1, the diagnostic accuracy of mpMRI for the detection of locally advanced disease is presented for all study years separately (radiologic learning curve). Over time, a negative trend was observed, although the differences in diagnostic accuracy were not statistically significant (sensitivity per year *p *= 0.12, specificity per year *p *= 0.57; sensitivity per half sample *p *= 0.61).

## Discussion

The preoperative assessment of local tumor stage affects important therapeutic decisions regarding radicality of the surgical procedure (i.e., a wide or less wide excision around the prostate; the performance of an ePLND) and the ability to preserve structures that relate to functional outcomes (i.e., to perform nerve-sparing surgery or not).

In our extensive, multicenter cohort, the accuracy of mpMRI for local tumor staging was evaluated. The reference standard was the pathology report following RARP. Intriguingly, more than half of the cases (55%) with locally advanced disease (pT3–4) remained undetected by preoperative mpMRI (see Table 2). Conversely, if mpMRI indicated locally advanced disease, in more than half of the cases, these results proved to be falsely positive, as pathological examination showed organ-confined disease. We observed no significant increase in diagnostic accuracy when confining the use of mpMRI to patients with high risk of locally advanced disease only.

Our results are in line with those observed in the review by De Rooij et al. [13]; and taking together, it appears questionable whether mpMRI is adequate to guide therapeutic decision-making. Moreover, mpMRI does not seem superior in predicting locally advanced disease compared to routine clinical parameters such as those used in the Partin tables [11]. There may be a role for mpMRI when results are combined with clinical parameters, as a recent study showed some increased accuracy for such combination (not a predefined nomogram) [23]. Future research might clarify if the incorporation of mpMRI to the established nomograms could increase diagnostic confidence.

Novel insights of this study include the clinical impact of erroneous staging and the evaluation of a radiologic learning effect. Radiologic understaging potentially impairs oncologic outcomes, as patients might not receive the indicated ePLND or might be operated with a possibly hazardous nerve-sparing approach. We have shown that in a third of all patients with locally advanced disease, radiologic understaging might have contributed to a form of undertreatment.

Radiologic overstaging, on the other hand, leads to patients unnecessarily being withheld nerve-sparing surgery, causing impaired potency and urinary continence. In our cohort, for 16 men with organ-confined disease, this form of overtreatment seemed present (6% of all patients with organ-confined disease).

The above-mentioned numbers should be interpreted cautiously. Due to the retrospective nature of our study, causality cannot be proven. Furthermore, no complete insight into surgical decision-making was present. For example, the choice to not perform an ePLND could have been caused by radiologic understaging, but patient factors outside the scope of this study may also have been the reason to omit an ePLND (e.g., a technically unfeasible procedure). The true extent of undertreatment, caused by inaccurate mpMRI findings, might, therefore, have been more limited.

The same applies the identified patients with potential overtreatment. We cannot know whether all of these patients would have opted for a nerve-sparing approach, if preoperative radiologic staging would have been correct. Preoperative erectile function, for example, may have been absent, rendering nerve-sparing surgery futile.

In our cohort, no radiological learning curve could be observed. Despite the rising experience with mpMRI and increasing number of performed procedures, the sensitivity for locally advanced disease did not improve over the years. In the previous reports as well, the number of included procedures had no influence on diagnostic accuracy [13], challenging the presumed gain of concentrating mpMRIs to a limited number of hospitals.

Although our sample size was substantial (430 patients), one might question whether 137 patients with locally advanced disease divided over different hospitals, over multiple years’ time, were sufficient to facilitate learning. It is important to realize, however, that radiologists were exposed to more mpMRI studies than included in this analysis (i.e., also radiotherapy patients are staged with mpMRI). Besides, the included centers are all recognized reference centers for PCa care, meaning that these numbers comprise the realistic clinical volumes in the present-day oncologic care.

In this study, we examined the existence of a radiologic learning curve on a hospital level. We cannot formally exclude the possibility that a learning curve was present for individual radiologists, as no strict, prospective protocol was followed. However, it is frequently stated that mpMRI should be confined to expert centers [3, 24], implying general hospital-level learning. As individual radiologists may change employment, the evaluation of a learning curve irrespective of changes in individual expertise is warranted.

Our study has some additional limitations. The available information on the radiologists performing mpMRI interpretation was limited. In the included hospitals, prostate mpMRIs are strictly reviewed by dedicated radiologists. Although this is widespread practice, we cannot formally exclude the possibility that less experienced radiologists have occasionally reviewed scans in referring centers. Standardly performing a central revision of the mpMRI images might have overcome such imperfection, but is not in line with the current clinical practice.

Another point is the impact of the rising number of scans on clinical workflow. We hypothesized that such increase would lead to more radiologic experience and thereby to higher diagnostic accuracy. However, we do not know that the available time radiologists were given to review all scans. Given the marked rise in number of scans, the available time per scan could have been comprised, possibly offsetting a potential learning effect.

The strength of our analysis lies in the fair representation of clinical reality in the present-day oncologic centers. If the volumes, methods, and interpretation of mpMRI acquisitions in these acknowledged reference centers do not lead to accurate results, it seems challenging to ensure widespread value of the imaging technique.

## Conclusions

In this multicenter, real-life clinical cohort of patients, the diagnostic accuracy of mpMRI for the detection of locally advanced PCa stages was limited. Therefore, it remains questionable whether the diagnostic accuracy of mpMRI is sufficient to guide therapeutic decision-making. In addition, no significant radiologic learning curve for the detection of locally advance disease was observed.
